# Immunological and Functional Characterization of RhoGDI3 and Its Molecular Targets RhoG and RhoB in Human Pancreatic Cancerous and Normal Cells

**DOI:** 10.1371/journal.pone.0166370

**Published:** 2016-11-10

**Authors:** Mercedes Piedad de León-Bautista, Maria del Carmen Cardenas-Aguayo, Diana Casique-Aguirre, Manuel Almaraz-Salinas, Sara Parraguirre-Martinez, Angelica Olivo-Diaz, María del Rocío Thompson-Bonilla, Miguel Vargas

**Affiliations:** 1 Departamento de Biomedicina Molecular, Centro de Investigación y de Estudios Avanzados del Instituto Politécnico Nacional, Avenida Instituto Politécnico Nacional 2508, col. San Pedro Zacatenco, C.P. 07360, Mexico City, Mexico; 2 Departamento de Fisiología, Facultad de Medicina, Universidad Nacional Autónoma de México, Avenida Universidad 3000, Col. Copilco Universidad, Delegación Coyoacán, C.P. 04510, Mexico City, Mexico; 3 Facultad de Bioquímica, Instituto Tecnológico de Milpa Alta, Independencia Sur 36, San Salvador Cuauhtenco, Milpa Alta, 12300, Mexico City, Mexico; 4 Departamento de Anatomía Patológica, Hospital General Doctor Manuel Gea González, Av. Calzada de Tlalpan 4800, Tlalpan, Sección XVI, 14080, Mexico City, Mexico; 5 Departamento de Biología Molecular e Histocompatibilidad, Hospital Doctor Manuel Gea González, Av. Calzada de Tlalpan 4800, Tlalpan, Sección XVI, 14080, Mexico City, Mexico; 6 Investigación Biomédica y Traslacional, Laboratorio de Medicina Genómica, Hospital 1° de Octubre, ISSSTE, Av. Instituto Politécnico Nacional No. 1669, Colonia: Magdalena de las Salinas, Delegación: Gustavo A Madero, 07760, Mexico City, Mexico; University of Nebraska Medical Center, UNITED STATES

## Abstract

RhoGDI proteins have been implicated in several human cancers; changes in their expression levels have shown pro- or anti-tumorigenic effects. Pancreatic Ductal Adenocarcinoma (PDAC) is a complex pathology, with poor prognosis, and most patients die shortly after diagnosis. Efforts have been focused on understanding the role of RhoGDI's in PDAC, specially, RhoGDI1 and RhoGDI2. However, the role of RhoGDI3 has not been studied in relation to cancer or to PDAC. Here, we characterized the expression and functionality of RhoGDI3 and its target GTPases, RhoG and RhoB in pancreatic cell lines from both normal pancreatic tissue and tissue in late stages of PDAC, and compared them to human biopsies. Through immunofluorescences, pulldown assays and subcellular fractionation, we found a reduction in RhoGDI3 expression in the late stages of PDAC, and this reduction correlates with tumor progression and aggressiveness. Despite the reduction in the expression of RhoGDI3 in PDAC, we found that RhoB was underexpressed while RhoG was overexpressed, suggesting that cancerous cells preserve their capacity to activate this pathway, thus these cells may be more eager to response to the stimuli needed to proliferate and become invasive unlike normal cells. Surprisingly, we found nuclear localization of RhoGDI3 in non-cancerous pancreatic cell line and normal pancreatic tissue biopsies, which could open the possibility of novel nuclear functions for this protein, impacting gene expression regulation and cellular homeostasis.

## Introduction

Pancreatic Ductal Adenocarcinoma (PDAC) is one of the most lethal cancers worldwide, in the USA, more than 48,960 new cases of PDAC occurred in 2015, with an estimated of 40,560 deaths: 19,850 female and 20,710 male [1]. The high number of cases could be due to the fact that PDAC is usually diagnosed at late stages, once it has disseminated, leading to poor prognosis and low survival rate. Many molecules have been implicated in the processes of dissemination, invasion and metastasis, including Rho GTPases, which are key components of the actin cytoskeleton reorganization [2]. Rho GTPases act like molecular switches: they have an inactive, GDP-bound stage and an active stage in which GDP is replaced with GTP. This cycle is highly regulated by three different groups of proteins: GEFs (guanine nucleotide exchange factors), GAPs (GTPase-activating proteins), and GDIs (guanine nucleotide dissociation inhibitors) [3]. In addition to mutations, overexpression and downregulation of Rho GTPases have been reported [4, 5], suggesting that this signaling pathway might be altered by molecules that regulate Rho GTPases, i.e., RhoGDIs.

It has been more than 20 years since Ohga and Fukumoto reported a regulator molecule for the RhoB GTPase that inhibits the GDP dissociation of this protein, known as a GDI [6, 7]. Currently, three different molecules have been described as belonging to the RhoGDI family: RhoGDI1, RhoGDI2 and RhoGDI3. RhoGDI1 is found ubiquitously and can interact with several GTPases [8]. The RhoGDI2 protein shares a 70.7% identity with RhoGDI1; at first, RhoGDI2 was described as expressed specifically in lymphoid and myeloid tissues, predominantly in B and T lymphocytes [9, 10], however, it is now known to be expressed in other tissues, including the brain, prostate and pancreas. RhoGDI3, the third protein in the RhoGDI family, shares 62.1% and 70.7% identity with RhoGDI1and RhoGDI2 respectively. It was first identified in murine cerebral tissue and was later found in human cerebral and pancreatic tissue. Unlike the other two RhoGDIs, RhoGDI3 contains an LDXXEL motif that confers anchorage into the membranes of Golgi vesicles [11–13].

Canonically, GDIs modulate the movement of Rho GTPases between the cytoplasm and plasma membrane by sequestering them in the cytosol, thereby protecting them from degradation. Removing Rho GTPases from the membrane maintains their inactive state [14–16]. In addition to its physiological functions, RhoGDI proteins have been implicated in several human cancers such as breast [17, 18], ovarian [19], myeloid leukemia [20] and liver cancers [21]. Changes in RhoGDI expression levels have shown pro- or anti-tumorigenic effects that depend on the cell type and tissue. PDAC is a challenging pathology to address, since most of the patients diagnosed die shortly after the diagnosis. Many researchers are focusing in understanding the possible role of RhoGDI proteins in PDAC. In 2009, it was reported that TrkBT1 sequesters RhoGDI1, leading to over-proliferation and increased metastatic potential through the hyperactivity of RhoA GTPase [22]. During the same year, it was demonstrated that the silencing of RhoGDI2 protein in pancreatic cancer cell lines caused the loss of the high capacity for neural invasiveness [23]. The role of RhoGDI3 has been poorly understood. However, in 2003, RhoGDI3 hyper-immunoreactivity was reported for normal breast tissue but not for cancerous breast tissue. These findings correlated with the mRNA expression of RhoGDI3, suggesting that this protein could be an important factor for clinical staging or use as a prognostic marker [24]. The role of RhoGDI3 in PDAC remains elusive and has not been studied in pancreatic cells. Considering that RhoGDI3 has tissue-specific expression, and that the regulation of GTPases by RhoG and RhoB, is involved in invasion, migration and tumor suppression, we decided to address its possible role in the progression of PDAC. Here we report the pattern of expression and functionality of RhoGDI3, and its targets RhoG and RhoB proteins in normal and cancerous pancreatic cells of different stages of the disease. Most importantly, we show that RhoGDI3 is located in the nuclei of normal pancreatic cell lines and human pancreatic tissue, suggesting a novel nuclear function for this protein, impact gene expression regulation and cellular homeostasis. Our results points to the importance of RhoGDI3 as a marker of progression and/or aggressiveness of PDAC.

## Materials and Methods

### Ethics Statement

This work complies with the current health laws of Mexico and was approved by the Ethics and Research Committees of the Hospital General Dr. Manuel Gea Gonzalez.

### Cell Culture

The cells were cultured at 5% CO_2_ and 95% of Atmospheric air at 37°C. The cell lines hTERT-HPNE (ATCC^®^ CRL-4023^™^, Manassas, VA), BxPC3 (ATCC^®^ CRL-1687^™^, Manassas, VA) and PANC-1 (ATCC^®^ CRL-1469^™^, Manassas, VA) were cultured in the conditions recommended by the supplier. All the cell lines were used between passages 2–14.

### Antibodies

We used the following antisera: mouse mAbs against RhoGDI3 (H95, sc-367757, Santa Cruz, CA), RhoGDI2 (sc-365663, Santa Cruz, CA), RhoGDI3 (E10, sc-365663, Santa Cruz, CA), RhoG (1F B3 E5, sc-80015, Santa Cruz, CA) and RhoB (sc-8048, Santa Cruz, CA), 58K Golgi protein (Ab27043, Cambridge, MA); rabbit pAbs against RhoGDIgamma (MBS710991, San Diego, CA), Rac1 (Cytoskeleton GL07, Denver, CO), Histone H3 (Ab1791, Cambridge, MA), Aldolase B (ab75751, Cambridge, MA). The secondary antibodies used were conjugated to horseradish peroxidase (anti-rabbit, Invitrogen 626520; anti-mouse, 656120, Waltham, MA) or to Alexa Fluor 488 (anti-rabbit and anti-mouse, Molecular probes A21057, A11034, Waltham, MA) or Alexa Fluor 680 (anti-mouse, Molecular probes A21057, Waltham, MA).

### Western Blotting

Western blots of protein samples were performed from monolayers of the cell lines hTERT-HPNE, BxPC3, and PANC-1. The cells were lysed in RIPA buffer containing 1X protease inhibitor cocktail (Roche 11873580001, Indianapolis, IN) and 1X phosphatase inhibitor cocktail (Roche 49068450001, Indianapolis, IN). Protein concentration was determined using the Lowry assay (DC Protein assay, BioRad 500–0114, Hercules, CA), 20 μg of protein were separated by 12% SDS-PAGE and transferred to PVDF membranes (Millipore IPVH00010, Billerica, MA) at 200 mA for 2 hours at 4°C. After blocking in 5% nonfat dry milk with TBS and 0.1% Tween 20, the membranes were incubated overnight at 4°C in the same buffer adding primary antibodies: anti-RhoGDIgamma (1:1000), anti-RhoGDIbeta (1:500), anti-RhoGDI3 (1:200), anti-RhoG (1:200), anti-RhoB (1:200), anti-Rac1 (1:500), anti-Histone H3 (1:15,000), or anti-Aldolase B (1:5000). Next, the membranes were washed 4 times with TBS-0.1% Tween 20 and then incubated with HRP-conjugated secondary antibodies (anti-rabbit, 1:6000; anti-mouse, 1:1000) for 1 hour at 37°C. Membranes were washed with TBS 3 times at 37°C. Protein bands were visualized on a ChemiDoc^™^ MP Imaging System (Bio-Rad, Hercules, CA) using SuperSignal^™^ West Pico Chemiluminescent Substrate (Thermo Scientific 34079, Waltham, MA).

### Immunofluorescence (IF) and Confocal Microscopy Imaging of Pancreatic Cell Lines

hTERT-HPNE, BxPC3 and PANC-1 cells were grown on coverslips (Tedpela 26020, Redding, CA) coated with poly-D-Lysine, washed twice with PBS, and then fixed with 4% PFA for 20 min. After a second wash, the cells were permeabilized with 0.1% Triton X-100 for 3 minutes at 37°C. Nonspecific binding was prevented using blocking buffer containing PBS-BSA 1% (BSA was fraction V, IgG free, SIGMA, St. Louis, MO). Cells were incubated with primary antibodies (anti-RhoGDIgamma (1:120), anti-RhoG (1:100), anti-RhoB (1:100), anti-58k (1:500) overnight at 4°C. The next day, the cells were washed twice with PBS-BSA 1% and then incubated with appropriate secondary antibodies (anti-rabbit conjugated with Alexa Fluor 488 (1:500) and anti-mouse conjugated with Alexa Fluor 680 (1:300)). Nuclei were stained with DAPI.

Immunofluorescence staining of cells stimulated with EGF was performed as described previously with some modifications [25]. Briefly, hTERT-HPNE, BxPC3 and PANC-1 cells were plated on coverslips at a density of 1 × 10^4^ cells per coverslip. After 24 h, cells were starved in DMEM/0.5% bovine serum albumin (BSA) for 6 hours. The medium was replaced with DMEM/0.5% BSA supplemented with recombinant human (rhEGF) (G502A, SIGMA, St. Louis, MO) to a final concentration of 100 ng/ml for periods of 0, 2 and 10 min. Cells on coverslips were treated as previously described. F-actin was labeled with rhodamine phalloidin (Invitrogen R415, Carlsbad, CA) for 1 hour. Images were captured using an Olympus laser-scanning confocal microscope FV-300 (Melville, NY) with 60X and 100X objectives. Overlap index was calculated by Pearson’s coefficient of colocalization (PCC), using the software FluoView 300 for Olympus laser-scanning confocal microscope FV-300 (Melville, NY). These calculations allow us to discover whether different labeled structures were present in the same region of the cell. PCC values range from 1 for two images whose fluorescence intensities were perfectly, linearly related, to −1 for two images whose fluorescence intensities were perfectly, but inversely, related to one another. Values near zero reflect distributions of probes that are uncorrelated with one another [26].

### Subcellular Fractionation

Separation of nuclear and cytosolic fractions of hTERT-HPNE, BxPC3 and PANC-1 cells was performed according to the methods previously described [27]. Briefly, cells were treated with rhEGF, followed by the addition of 500 μl of fractionation buffer (250 mM sucrose, 20 mM HEPES pH 7.4, 10 mM KCl, 1.5 mM MgCl2, 1 mM DTT, 1X protease inhibitor cocktail and 1X phosphatase inhibitor cocktail) to the monolayers. Cells were collected, homogenized by 10 passages through a 25 G needle, and incubated on ice for 30 min. The nuclear pellets were extracted by centrifugation at 720 ×g for 5 min. The supernatants were resuspended in Laemmli buffer, corresponding to the cytosolic fraction. The nuclear pellets were washed once by adding 500 μl of fractionation buffer, dispersed with a pipette, passed through a 25 G needle 10 times, and centrifuged again at 720 ×g for 5 min. The wash buffer was removed, and the nuclear pellets were sonicated briefly (3 s) on ice. Protein concentrations were determined using the Lowry assay. Nuclear and cytosolic fractions were resuspended in 2X Laemmli buffer. Then, cell lysates (20 μg) were separated by SDS-PAGE on 15% gels. Proteins were transferred to PVDF membranes and analyzed via Western blotting. Anti-histone H3 antibody was used as a nuclear marker and anti-Aldolase B antibody was used as a cytosol marker.

### RhoG and RhoB Activation Pulldown Assays

RhoG activity was evaluated using a pulldown assay with the protein ELMO1 expressed in the E. coli strain DH5-α as a fusion protein with GST (plasmid kindly donated by Dr. Michael Schnoor). Bacterial pellets were suspended in PBS and DTT and broken by sonication. After centrifugation at 10,000 rpm for 15 minutes at 4°C, the supernatants were resuspended in Triton 10%. GST-ELMO1 protein was purified by incubation with glutathione beads (GE Healthcare Bio-Sciences, Uppsala, Sweden) for 1 hour followed by 3 washes in cold lysis buffer.

Once hTERT-HPNE, BxPC3 and PANC-1 cells were treated with rhEGF as previously described for immunofluorescence staining, the cells were lysed with cold lysis buffer containing 1% Triton, 150 mM NaCl, 50 mM Tris pH 7.4, 1X Roche Complete protease inhibitor and 1X Phostop cocktails in order to measure RhoB and RhoG activity. Lysates were clarified by centrifugation at 10,000 x g and 4°C for 1 min, after which they were collected and placed on ice. Measurements of protein concentrations in the lysates were performed using Lowry assay.

For nucleotide loading of endogenous RhoG and RhoB proteins, 400 μg of lysate were incubated with 10 mM EDTA and 10 mM GTPγS for 15 min at 30°C with gentle shaking. To stop the reaction, MgCl_2_ was added to a final concentration of 60 mM. Reactions were incubated with GST-ELMO beads (50 μg) and Rhotekin-RBD protein (Cystoskeleton RT02, Denver Co) beads (50 μg) for 1 h at 4°C on a rotator and then washed 3 times with lysis buffer. The samples were eluted with 2X Laemmli buffer, separated by 15% SDS-PAGE, transferred to PVDF membranes, and analyzed by Western blotting using anti-RhoG, anti-RhoB, anti-Rac-1 and anti-GAPDH antibodies.

### Immunofluorescence on Paraffin-Embedded Sections from Human Pancreatic Biopsies

We analyzed the following paraffin-embedded samples: 3 biopsies from normal pancreas and 3 from PDAC, that were obtained from the pathology service of the Hospital General Dr. Manuel Gea Gonzalez, Mexico City. This work complies with the current health laws of Mexico and was approved by the Ethics and Research Committees of the Hospital General Dr. Manuel Gea Gonzalez. The samples were processed as follows: 5 μm sections were obtained and mounted on pre-charged slides (Am-Labs-1500, Boulder, CO) The slides were deparaffinized and rehydrated: Xylene-ethanol 1:1 (3 min), 100% ethanol (3 min), 95% ethanol (3 min), 70% ethanol (3 min), 50% ethanol (3 min). Heat-induced epitope retrieval was performed using sodium citrate (pH 6.0) and boiling in a pressure cooker for 5 minutes. The immunohistochemical staining protocol was performed as described for IF confocal microscopy on pancreatic cell lines.

## Results

### RhoGDI3 Expression Pattern in Normal and Cancerous Human Pancreatic Cells Lines

**Fig 1:**
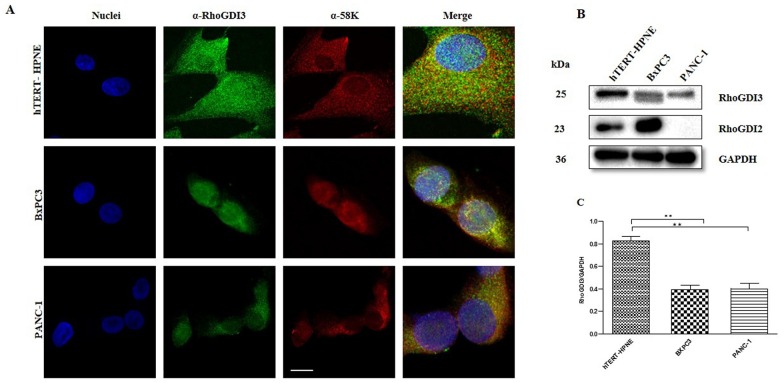
Cancerous and non-cancerous pancreatic cell lines show different expression patterns of RhoGDI3 protein. (A) Immunofluorescence microscopy analysis of RhoGDI3 protein (green); 58 kDa protein, Golgi apparatus marker (Red) and Nuclei (DAPI, blue) was performed on hTERT-HPNE (upper panel), BxPC3 (middle panel) and PANC-1 (bottom panel) cells lines. (B) Representative Immunoblot using antibodies anti-RhoGDI3, anti-RhoGDI2 and anti-GAPDH were used as loading control. Total lysates from hTERT-HPNE, BxPC3 and PANC-1 cell lines were analyzed. (C) Densitometric analysis of the bands detected in the Western blots of RhoGDI3 (n = 3) of protein extracts from all three cell lines, the data was normalized to GAPDH. Densitometric analysis was determined with Image Lab software. Values are means ± SEM, **P<0.005 (Anova-test). Scale bar 20μm.

RhoGDI3 protein is the only member of the RhoGDI family known to be partially associated to the Golgi apparatus in addition to its cytosolic localization [13]. We analyzed the subcellular localization of RhoGDI3 in non-cancerous (hTERT-HPNE) and cancerous (BxPC3 and PANC-1) human pancreatic cell lines by immunofluorescence and confocal imaging. BxPC3 represents an early stage of the disease and PANC-1 corresponds to a more aggressive stage of this cancerous disease. We observed that RhoGDI3 co-localizes with the 58 kDa protein, a Golgi marker, in all three cell lines, showing a punctate pattern, that suggests its vesicular localization (Fig 1). Notably, RhoGDI3 had a diffuse pattern of expression in hTERT-HPNE cells, in contrast to BxPC3 and PANC-1 cells, where the protein was mostly located around the nuclei, colocalizing with the 58k Golgi marker (Fig 1A).

There are many reports about the overexpression or downregulation of RhoGDI1 and RhoGDI2 proteins in many types of cancers, with contrasting results [17, 20, 24, 28–31], but there is a lack of information about the expression of RhoGDI3 in pancreatic cancer. Thus, to complement the immunofluorescence analysis of RhoGDI3 expression in the pancreatic cell lines, we carried out an immunoblot analysis of total protein extracts using anti-RhoGDI3 antibody, detecting a band of the expected molecular size of 25 kDa, this corresponds to the predicted size for RhoGDI3 (Fig 1B). We found significantly lower RhoGDI3 expression levels (about half of the level) in the extracts from BxPC3 and PANC-1 cell lines as compared to the extracts of control non-cancerous cell line hTERT-HPNE (Fig 1C). Notably, in the BxPC3 extracts, we observed a second band at ~23 kDa detected with the antiserum to RhoGDI3. Therefore, we investigated whether the anti-RhoGDI3 antibody might cross-react with RhoGDI2 or whether it is a specific band of a potential isoform of RhoGDI3, or a processed form of RhoGDI3. The same PVDF membrane was incubated with an anti-RhoGDI2 antibody, and we observed that RhoGDI2 antibody detects a 23 kDa band, and it does not cross-react with RhoGDI3; furthermore, we found that RhoGDI2 is not expressed in PANC-1 extracts (Fig 1B). We could not discard that the 23 kDa band detected with anti-RhoGDI3 antibody in the BxPC3 extracts could be a product of protein processing.

### Altered Expression Levels of RhoG and RhoB in Cancerous Human Pancreatic Cell Lines

**Fig 2:**
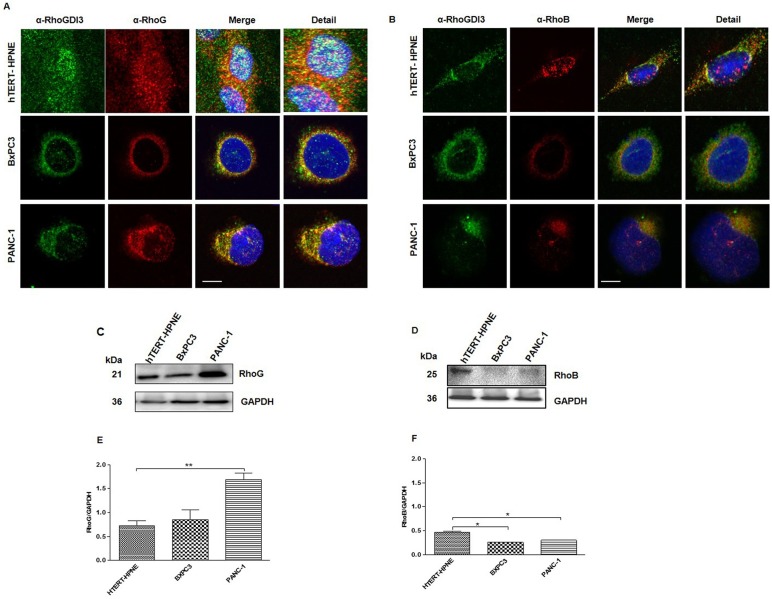
The expression of RhoG and RhoB proteins is altered in cancerous pancreatic cell lines. Immunofluorescence microscopy analysis of RhoGDI3 protein (green); RhoG (A) and RhoB (B) (Red) and Nuclei (DAPI, blue) of hTERT-HPNE (upper panel), BxPC3 (middle panel) and PANC-1 (bottom panel) cells lines. Representative Immunoblot using antibodies anti-RhoG (C), anti RhoB (D), GAPDH was used as loading control. Total lysates from hTERT-HPNE, BxPC3 and PANC-1 cell lines were analyzed. Total amount of RhoG (E) and RhoB (F) proteins was normalized to GAPDH (n = 3). Immunoblot densitometric analysis was performed with Image Lab software. Values are means ± SEM, **P<0.005, *P<0.005 (Anova-test). Scale bar 10μm.

To determine whether RhoG colocalized with RhoGDI3 in hTER-HPNE, BxPC3 and PANC-1 pancreatic cells grown in the standard medium, immunofluorescence and confocal microscopy studies were carried out. We observed RhoG protein expression in a punctate pattern in all cell lines (Fig 2A), although the subcellular colocalization with RhoGDI3 was different among the three cell lines. In hTERT-HPNE cells, the two proteins presented similar distributions, with each protein expressed throughout the whole cell in a diffuse pattern (Fig 2A upper panel). In contrast, the two proteins were perinuclear localized (Fig 2A middle and bottom panel) BxPC3 and PANC-1 cells, consistent with its previously reported localization [13].

Using immunoblot assays with antisera to RhoG, we detected a 21 kDa band, corresponding to the molecular weight of RhoG (Fig 2C). This band was 3-fold overexpressed in PANC-1 but not in BxPC3 extracts, as compared to control non-cancerous cells (hTERT-HPNE) (Fig 2E).

Although pull-down assays have shown that RhoB interacts with RhoGDI3 [12], this interaction has not been demonstrated *in vivo*. Using an immunofluorescence assay, we found colocalization of RhoB and RhoGDI3 in a diffuse pattern in hTERT-HPNE and BxPC3 cells (Fig 2B upper and middle panel). In contrast, PANC-1 cells exhibited a more polarized pattern in which RhoGDI3 and RhoB were colocalized (Fig 2B bottom panel). Accordingly, RhoB expression was very low in BxPC3 and PANC-1 cells as compared to control non-cancerous cells hTERT-HPNE (Fig 2D and 2F).

### Functional Analysis of the Activation stage of RhoG GTPase in hTERT-HPNE, BxPC3 and PANC-1 Human Pancreatic Cell Lines

**Fig 3:**
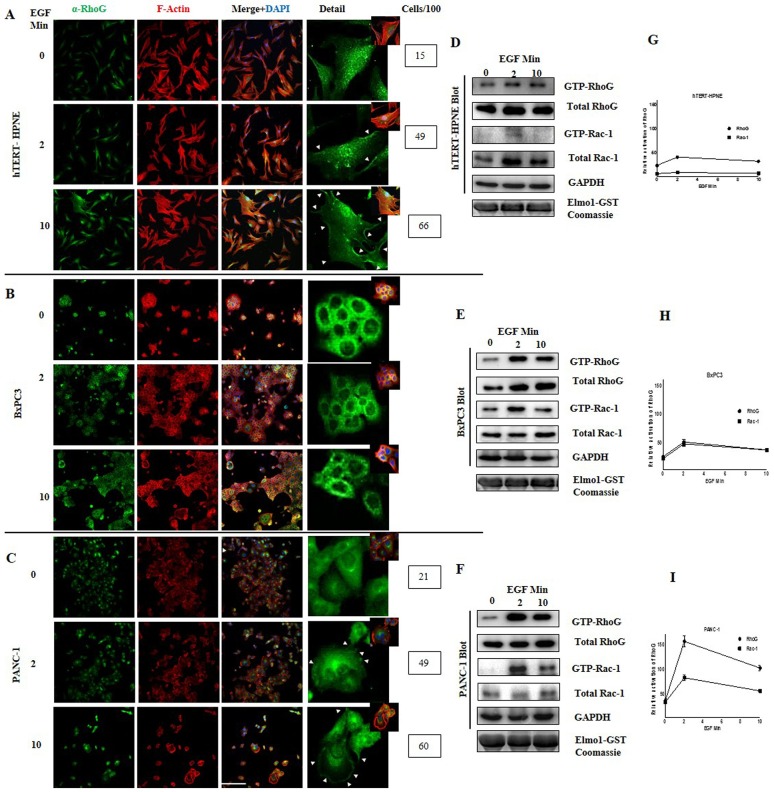
Activation of RhoG GTPase in hTERT-HPNE, BxPC3 and PANC-1 pancreatic cell lines. Cells were starved 6 hours and confronted with rhEGF for the period of 0, 2 and 10 minutes (Marked as 0, 2 and 10). Fluorescence microscopic staining of RhoG (green) was carried out in hTERT-HPNE (A), BxPC3 (B) and PANC-1 (C) cells lines. To show the cytoskeleton reorganization, F-Actin was stained with rhodamine phalloidin. Measurement of RhoG activity was performed using RhoG pulldown assay. Immunoblots for RhoG, Rac-1 and GAPDH proteins for hTERT-HPNE (D), BxPC3 (E) and PANC-1 (F) are shown. To quantify the amount of RhoG-GTP and bound-Rac-1 through the temporal course, densitometric analysis was performed using Image Lab software, hTERT-HPNE (G), BxPC3 (H) and PANC-1 (I). For comparison of RhoG activity, the total amount of RhoG in cell lysates was normalized to total RhoG. GAPDH was used as a protein loading control. ELMO1-GST beads coomassie are shown as beads loading control. Arrowheads denote the localization of RhoG into the peripheral membrane; boxes with number represent the number of cells with this phenotype. Scale bar 100 μm.

Using immunelabeling with antiserum to RhoG and confocal microscopy imaging, we analyzed cell morphology and RhoG localization after rhEGF stimulation. To confirm the actin-cytoskeleton rearrangement after treatment, the cells were stained with rhodamine phalloidin. Stimulated hTERT-HPNE and PANC-1 cells showed two patterns: RhoG relocated to the lamellipodial protrusions and to the cytosol (Fig 3A and 3C). While BxPC3 cells did not show a clear relocalization of the protein at any time point studied (Fig 3C), it reminds in a diffuse pattern of expression, regardless RhoG activation state. RhoG-GTP expression levels in hTERT-HPNE, BxPC3 and PANC-1 cell lines at 0, 2 and 10 minutes (T0, T2, and T10) after rhEGF exposure were evaluated by using a RhoG activation pull-down assay. We observed a basal activation of RhoG in all three cell lines at T0, that raises over time, reaching a peak at T2 and decreasing at T10 (Fig 3D, 3E and 3F). However, the pancreatic cancerous cell lines BxPC3 and PANC-1 showed a greater rise of RhoG activation at T2 as compared to hTERT-HPNE cells (Fig 3F and 3G). We quantified the number of cells in which RhoG relocated to the periphery or near the plasma membrane after rhEGF treatment. We observed that localization to the plasma membrane is mostly present at T2 throughout T10 in hTERT-HPNE and PANC-1, whereas this rearrangement is almost absent at T0, T2 and T10 in BxPC3cells (Fig 3 boxes and arrowheads).

In 2003, Katoh et al. reported that RhoG-GTP activates Rac1 via ELMO/Dock180 [32]; therefore, we were able to use the same RhoG activation pulldown assay to investigate Rac1-GTP. We found that Rac-1 is present at the three times tested (T0 to T10), with significant expression at T2 and declining activation at T10 in all three cell lines, with particularly higher expression in BxPC3 and PANC-1(Fig 3F and 3G).

### Differential Activation of RhoB GTPase in PDAC Cell Lines

**Fig 4:**
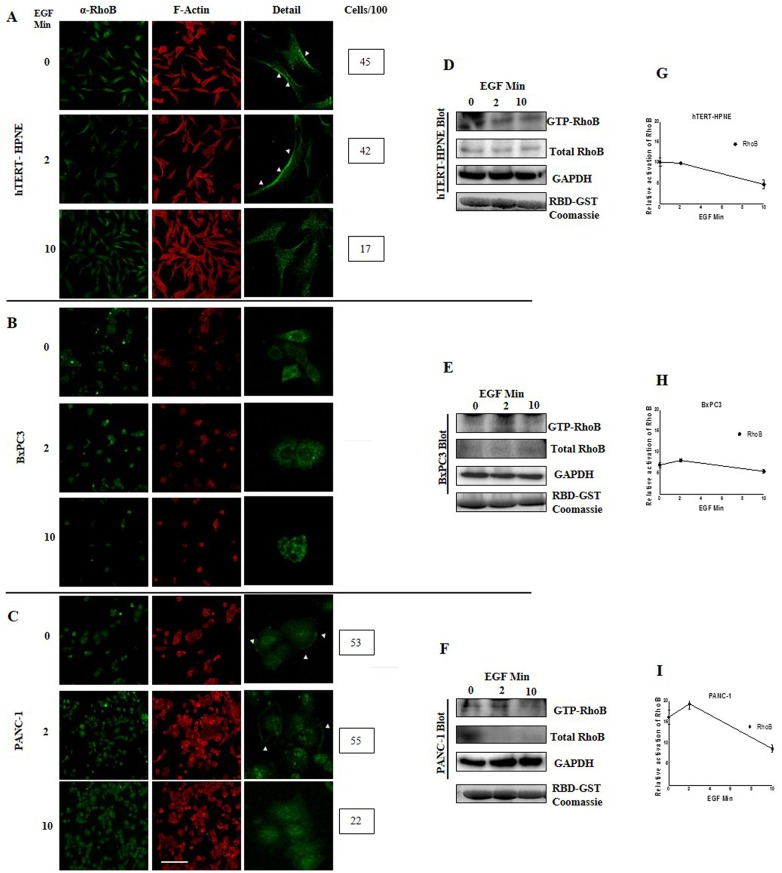
GTPase RhoB shows differential activation in PDAC cell lines. Cells were starved for 6 hours and treated with rhEGF for a period of 0, 2 and 10 minutes (Marked as 0, 2 and 10 min). An immunofluorescence microscopy analysis of RhoB (green) was carried out on hTERT-HPNE (A), BxPC3 (B) and PANC-1 (C) cells lines. To show the cytoskeleton reorganization, F-Actin was stained with rhodamine phalloidin. Measurement of RhoB activity was performed using RhoB pulldown assay. Immunoblots for RhoB and GAPDH, as loading control for hTERT-HPNE (D), BxPC3 (E) and PANC-1 (F) cell lines are shown. To quantify the amount of RhoB-GTP, densitometric analysis (n = 3) was performed using Image Lab software for samples of hTERT-HPNE (G), BxPC3 (H) and PANC-1 (I) cell lines. For comparison of RhoB activity, GTP-RhoB was normalized to total RhoB. GAPDH was used as a protein loading control. Coomassie of RBD-GST beads are shown as beads loading control. Arrowheads denote the localization of RhoB into the peripheral membrane; boxes with number represent the quantity of cells per field with this phenotype. Scale bar = 100 μm.

Although RhoB protein expression is almost absent in BxPC3 and PANC-1 cell lines, we wanted to investigated whether the RhoB protein is activated in those cells. Due to the fact that RhoB could be activate by stimulation with growth factors, we treated the cells with rhEGF at 0, 2 and 10 minutes (T0, T2, and T10) to evaluate the activation state of RhoB. Pull-down assays of hTERT-HPNE cells showed the activation of RhoB at T0 and T2, with decreasing activation at T10 (Fig 4A and 4D). However, BxPC3 cells showed no clear activation pattern (Fig 4B and 4E) after treatment with rhEGF, and PANC-1 cells displayed activation at T0 and T2 after treatment with rhEGF, similar to the normal cell line (hTERT-HPNE) (Fig 4C and 4F). We also quantified the numbers of cells in which RhoB was localized in the periphery or near the plasma membrane after rhEGF treatment, and observed that in hTERT-HPNE and PANC-1 cells, localization to the plasma membrane is present from T0 throughout T2 but decreased at T10 in hTERT-HPNE (Fig 4 boxes and arrowheads). This observation is in agreement with our results on the activation of RhoB.

### Dynamic Role of RhoGDI3 in Pancreatic Cell Lines

**Fig 5:**
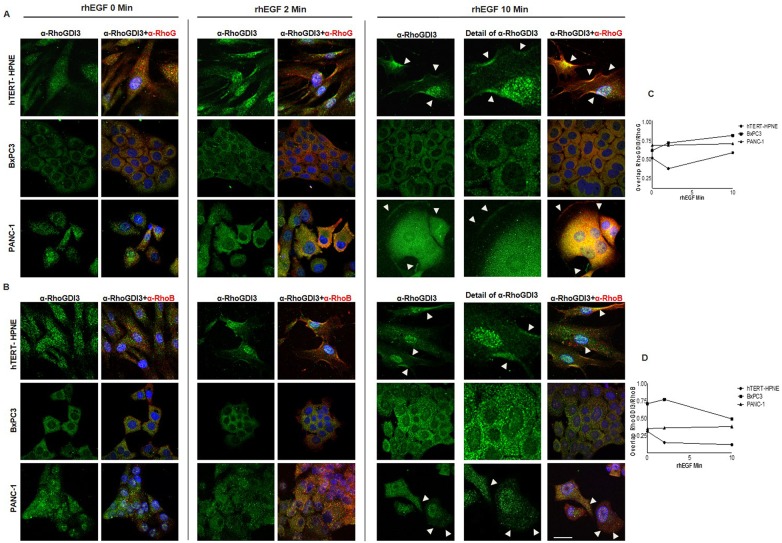
The dynamic role of RhoGDI3 in the pancreatic cell lines. Cells were starved for 6 hours and activated with rhEGF for the period of 0, 2 and 10 minutes (marked above the images as 0, 2 and 10 min). An immunofluorescence microscopy analysis of RhoGDI3 (green), RhoG (A) and RhoB (B) (red, not shown) was carried out on hTERT-HPNE (upper panel), BxPC3 (middle panel) and PANC-1 (bottom panel) cells lines, at the time point of 10 min it is shown the detail of RhoGDI3 staining to highlight the signal at the lamellipodial protrusions evident only in the cell lines hTERT-HPNE and PANC-1 (white arrowheads). The colocalization index was determined using the confocal Olympus software FluoView 300 for RhoGDI3 with RhoG and RhoB. Overlap index is shown in Fig 5C and 5D, respectively, index 1 corresponds to maximum overlap and cero corresponds to negative overlap. Scale bar 10 μm.

We next investigated the dynamics of the colocalization of RhoGDI3 and Rho GTPase proteins under rhEGF treatment. To that end, we carried out immunofluorescence and confocal microscopy imaging at the same 3 time points, T0, T2 and T10 min, using the anti- RhoGDI3, anti-RhoG and anti-RhoB antisera. The pattern of expression varies among the three cell lines analyzed. In hTERT-HPNE and PANC-1 cells, RhoGDI3 showed a relocalization from the perinuclear zone to the plasma membrane, specifically to lamellipodial protrusions, and this relocalization was more evident at T10 (Fig 5A and 5B, arrowheads, S6 Fig). Whereas, BxPC3 cells did not show a characteristic phenotype, the signal was diffuse. Next we investigated the possibility of colocalization with RhoG and RhoB, and found colocalization of these proteins in particular at the cellular protrusions (Fig 5C and 5D). One of the most remarkable findings was the enrichment of RhoGDI3 in the nucleus of hTERT-HPNE non-cancerous cells, but not in the nuclei of cancerous cells, despite comparable the cytosolic and nuclear distribution of RhoG in all three cell lines.

### Nuclear Targeting of RhoGDI3 in hTERT-HPNE Cells after rhEGF Treatment

**Fig 6:**
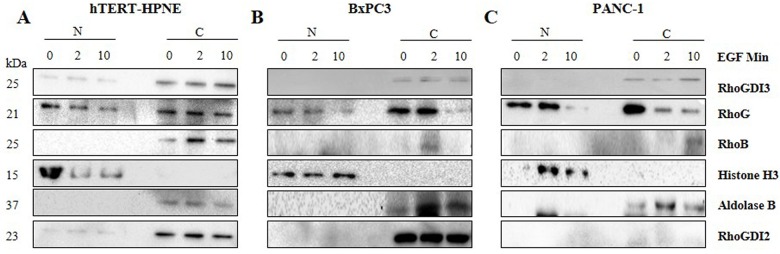
Nuclear localization of RhoGDI3 in rhEGF treated hTERT-HPNE cells. Subcellular fractionation was performed after cells were treated with rhEGF (marked above the images as 0, 2 and 10 rhEGF Min). Nuclear (N) and cytosolic (C) fractions from hTERT-HPNE (A), BxPC3 (B) and PANC-1 (C) cell lines were obtained and analyzed by immunoblotting, using anti-RhoGDI3, anti-RhoG, anti-RhoB antibodies. Anti-histone H3 antibody was used as a nuclear control and anti-Aldolase B antibody was used as a cytosol control. 20 μg of cell lysates were loaded.

To confirm our immunofluorescent finding of the nuclear localization of RhoGDI3 in hTERT-HPNE cells at T0, T2 and T10, we performed subcellular fractionation to obtain nuclear and cytosolic fraction and analyzed them by Western blotting. Cells were treated with rhEGF using the same conditions as for the immunocytochemistry experiments. The purity of the nuclear extracts was confirmed with an anti-histone H3 antibody, and no signal was detected with this antibody in the cytoplasmic fractions. As a marker for the cytoplasmic extracts we used an anti-Aldolase B antibody, and no signal was detected with this antibody in the nuclear fractions (Fig 6). Notably, we confirmed that RhoGDI3 was present in the nuclear fraction in hTERT-HPNE cells (Fig 6A) but not in the cancerous cell lines (PANC-1 and BxPC3), in which RhoGDI3 expression is limited to the cytosolic fraction. We also confirmed that RhoG is present in both, the nuclear and cytoplasmic fractions from all three cell lines. However, we founf that RhoB was not observed in the nuclei of any of the three pancreatic cell lines studied, which contradict reports from other cellular systems [33]. Furthermore, we observed a nuclear and cytosolic localization of RhoGDI2 in hTERT-HPNE, while the localization in BXPC3 was purely cytosolic. Finally, we confirmed that RhoGDI2 is absent in the PANC-1 even though the cytosolic protein was concentrated.

### Nuclear Localization of RhoGDI3 in Normal Human Pancreatic Tissue

**Fig 7:**
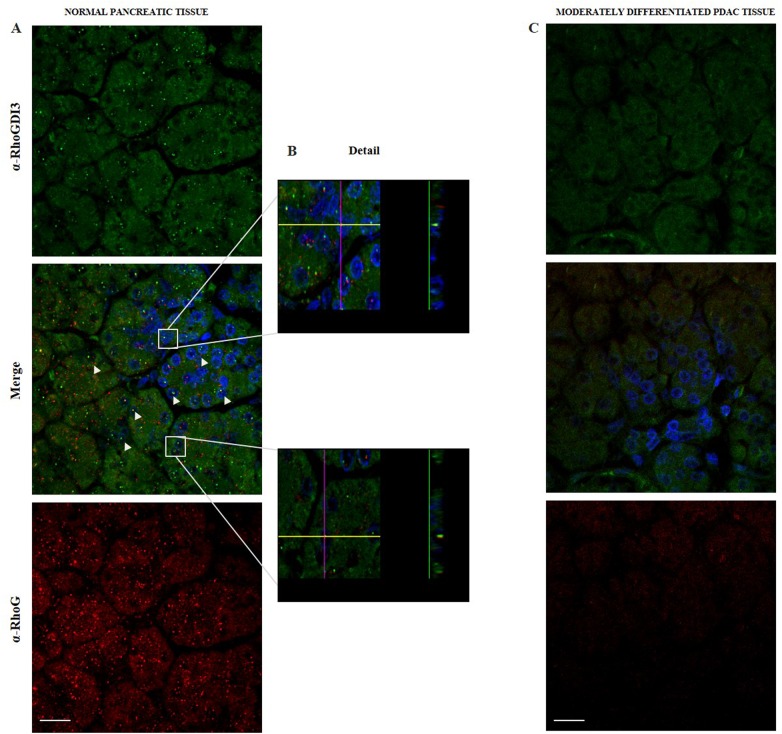
Nuclear localization of RhoGDI3 in normal pancreatic tissue. Immunofluorescence microscopy staining of RhoGDI3 (green) and RhoG (red) was carried out on human pancreatic normal (A) and moderate (aggressiveness) PDAC biopsies (C). (B) Magnification and lateral view of the immunofluorescence of RhoGDI3 and RhoG, to evidence nuclear localization in human pancreatic normal tissue. Arrowheads denote the localization of RhoGDI3 and RhoG into the nuclei. Scale bar 10 = μm.

The experiments described above demonstrate the presence of RhoGDI3 in the nuclei of hTERT-HPNE cells, a normal human pancreatic cell line Next we investigated whether RhoGDI3 is localized in the nuclei of cells from normal and cancerous human pancreatic biopsies. To this end, we carried out immunofluorescence microscopy on paraffin-embedded samples from human pancreatic biopsies, as described in the materials and methods. We observed that RhoGDI3 and RhoG proteins were primarily present in the cytoplasm in normal pancreatic tissue, with evident nuclear localization (Fig 7). However, we did not observe colocalization of RhoGDI3 and RhoG in normal pancreatic tissue, unlike our findings on pancreatic cell lines (Fig 1). Interestingly, although we did observe the presence of RhoGDI3 and RhoG in the nuclei (Fig 7A) of the normal human pancreatic tissue samples, RhoG protein expression was almost absent in the human PDAC tissue samples, with only minimal staining in the cytoplasm (Fig 7C). Interestingly, while in cancerous pancreatic cell linesRhoGDI3 protein expression is lost from the nuclei and it is only present in the cytosolic fractions; in non-cancerous cell lines, RhoGDI3 protein expression is present in both, cytoplasmic and nuclear fractions Furthermore, in normal pancreatic tissue, RhoGDI3 and RhoG where expressed in both cytosol and nuclei, however, these two proteins didn’t show any colocalization.

## Discussion

For the first time, we reported the different subcellular expression patterns and activation state of RhoGDI3 protein as well as the expression of two Rho GTPases, RhoG and RhoB, documented as target molecules for this negative regulator, in normal and cancerous human pancreatic cancer cell lines and human pancreatic tissue. Our findings could be useful to propose new markers of tumor progression and aggressiveness in PDAC. Canonically, RhoGDI proteins function in the cytosol, where they act like chaperones to solubilize Rho proteins and are mediators, monitoring the exchange of Rho proteins from protein complexes to cell membranes and vice versa. It was thought that they could have redundant functions, but their distinct expression patterns in different tissues and cell lines have suggested that they are not redundant; RhoGDI1 knockout mice, described by Towaga et al., is characterized by the development of kidney failure and infertility [34], whereas RhoGDI2 knockout mice showed a mild alteration in the oxidative state in phagocytic cells [35]. Due to the emerging role of RhoGDI’s proteins, RhoGDI1 and RhoGDI2, in many types of cancers [17, 20, 21, 36–38] and given the frequency with which those molecules expression is regulated in cancer, it is comprehensible that RhoGDI’s proteins have crucial roles in the carcinogenic process.

Similarly, to the findings of Jiang et al. in breast cancer, [24], we observed reduced expression of RhoGDI3 in both cancerous pancreatic cell lines BxPC3 and PANC-1, which correlates with the reduced expression of RhoGDI3 in breast cancer cells. Moreover, we analyzed the target small GTPases, RhoG and RhoB, and we found a clear reduction of RhoB in pancreatic cancerous cell lines, but not in non-cancerous pancreatic cells, that correlates with the fact that RhoB protein deletion promoted tumor formation [39]. Contrary to RhoB, RhoG protein was overexpressed only in PANC-1, a cell line that corresponds to the late stage of PDAC; whereas, the levels of RhoG in BxPC3 (cell line that corresponds to the early stages of PDAC) where no different from its levels in hTERT-HPNE (non-cancerous cell line). PANC-1 cells are described as a highly metastatic cell line, since they were isolated from adenocarcinoma in the head of pancreas, which invaded the duodenal wall and had generated metastases in one peripancreatic lymph node [40]. In order to metastasize, it is possible to speculate that a cell must overexpress molecules that control the movement such as RhoG, that might facilitate the displacement of the cell to other microenvironments. This statement correlates with the cell phenotype of BxPC3 cell line, a cell line obtained from a tumor circumscribed to the body of the pancreas in which no evidence of metastasis was found [41]. And, according to our results, RhoG expression is very low in this cell line. Despite the different levels of RhoG and RhoB in the three different cell lines, we found a similar but unequal activation state of these molecules, since the RhoG raises its activation at time 2 minutes and maintains its activations until time 10 minutes. This observation is supported by Rac-1 increased expression at time 2 min, due to the fact that when RhoG is activated by Trio-GEF in Golgi apparatus, this protein is driven to the cell periphery and once there, activated RhoG is able to induce the translocation of the ELMODock180 complex to the plasma membrane. This gives the possibility of multicomplex formation, which in turn, could activate Rac-1 and lead to formation of lamellipodia at the cell periphery [32]. When RhoGDI3 is transiently expressed in MDCK cells the activation of RhoG is hardly seen, suggesting the inhibitory dissociation activity [42]. Our findings revealed that the decreased expression of RhoGDI3 and the increased expression of RhoG proteins in PANC-1 cell line could not counteract the activation of RhoG, suggesting that the levels of RhoGDI3 where not enough to block the activation of RhoG. It has also been shown that the RhoGDI1 protein is an inhibitor of GEF proteins, which overlap the binding site GDI [43]. Moreover, the fast activation of RhoG in PANC-1 cell line in response to rhEGF at time 2 minutes, could be due to the overexpression of Epidermal Growth Factor Receptor (EGFR), which is characteristic in PANC-1 but not in BxPC3 cell lines [44]. Nevertheless, if this were the case, we should have seen a continuous activation of RhoG at time 10 minutes, but we observed a clear saturation of the system, in which the activation state was turned down, which could reinforce the idea that there are not enough inhibitory molecules that can keep inactivated the RhoG GTPase in the cytoplasm, giving place to the migration event through Rac-1 activated RhoG-dependent that could promote metastasis.

The RhoB is one of the downregulated GTPases in breast cancer [24] and invasive carcinomas of the head and neck [45]; accordingly, we found a decreased RhoB protein expression in the pancreatic cancer cell lines BxPC3 and PANC-1, as compared to non-cancerous pancreatic cell line hTERT-HPNE. Due to the role of RhoB as a tumor suppressor, that inhibits growth, cell migration, invasion and maintenance of mesenchymal morphology, functioning as a negative modifier in cancer, thus it is evident that a reduction in its level of expression could favor cancer progression [46] 2016; [47]. The subcellular localization of RhoB in hTERT-HPNE non-cancerous cell line diverges from the other two cancerous cell lines. In hTERT-HPNE it has a mostly homogeneous distribution; which could be due to the coexistence of two modified forms of RhoB: geranylgeranylated RhoB (RhoB-GG) and farnesylated RhoB (RhoB-F) [48], which are respectively localized in late endosomes and near cell membrane. RhoB GTPase is an unstable protein that is rapidly and transiently induced by a variety of stimuli, such as EGF [49, 50]. Our findings revealed a clear activation of RhoB at time 0 and 2 min in hTERT-HPNE and PANC-1 but not in BxPC3, coinciding with the presence of RhoB at the cell periphery, where it controls EGFR recycling [51], the gain of RhoB-GG postraslational modification, makes more efficiently this recycling event to the plasma membrane [48]. At time 10 minutes, we observed a clear redistribution of RhoB along the cytoplasm surrounding the nuclei, where it is proposed that activated Akt exhibits increased accumulation upon survival stimuli [33]. Our important finding on the activation of RhoB in PANC-1 cell line, could reflect a different cellular process that occurs in aggressive cancerous cells, PANC-1 metastatic, in which RhoB affects a basic pathway required for Rac-driven lamellipodium extension-stability [52]. Thus, RhoB might modulate the high migration rate in response to rhEGF treatment; nevertheless, more research is needed to elucidate this mechanism in cells where the expression of RhoB is apparently lost but could be induced under very specific stimuli, switching on signaling pathways involved in tumor progression.

Adra et al. reported the non-canonical nuclear localization sequence (NLS) found in RhoGDI3, suggesting that this protein might be targeted to the nuclei in a passive way [12]. We observed for the first time, the presence of RhoGDI3 in the nuclei of normal human pancreatic cell line hTERT-HPNE and normal human pancreatic tissue. This finding is supported by the fact that after RhoGDI2 has been cleaved by caspase 3, it is relocated to the nucleus, during apoptosis [53]. In accordance with this fact, we observed a clear localization of RhoGDI3 in the nuclei and the cytoplasm from hTERT-HPNE. Using RNAi methodology Lu et al. demonstrated that the loss of RhoGDI3 in neural cells induced changes in cell morphology, that were consistent with decreased transcription of genes like RhoA, Cdc42, Limk2, and N-WASP, molecules that impact the reorganization of the actin cytoskeleton [54]. Hence, this discovery could be related to homeostasis in normal but not in cancerous cells, at least in pancreatic cells, nevertheless, it is necessary to inquire what could be the functional role of nuclear RhoGDI3 in normal pancreatic cell.

We could not leave behind that RhoG GTPase was found in the nucleus off all three cell lines and normal pancreatic tissue, thus we investigated whether the RhoG GTPase has a NLS that could explain its nuclear localization. Using the cNLS [55] two predicted monopartite signals were found at 179 and 182 residues with 2 and 2.5 score respectively, both at the N-amino terminal. Until now, there is no previous evidence of a nuclear RhoG. Nevertheless, it is possible that RhoG is relocated into the nuclei after a starvation state, where it probably could play a role in the regulation of transcription factors. Currently it has been documented that RhoG promotes the transcriptional activation of Stat3 in murine fibroblasts and human cells equally as was reported for RhoA [56] and other GTPases [57]. Therefore, our finding of nuclear localization of RhoG could pave the road for a better understanding of alternative new functions of this protein that may contribute to carcinogenesis.

Our results suggest that the levels of RhoG and RhoB GTPases and their negative regulator RhoGDI3 might be linked to the aggressiveness of the pancreatic cancerous cell lines. It is possible that RhoGDI3 could induce the downregulation of RhoG and RhoB. In this respect, one of the canonical functions of RhoGDI’s is to protect the free prenylated cytosolic Rho GTPases against the degradation by the proteasome. We propose that these three proteins can be considered markers of aggressiveness in PDAC, however more research is warranted, in order to elucidate their possible implications in normal, inflammatory, and cancerous human samples.

## Supporting Information

S1 Fig RhoGDI3 recruits RhoG in the three pancreatic cell lines. Lysates from the three cell lines were immunoprecipitated (IP) with anti-RhoGDI3 and unrelated antibody, (A) Coomassie blue staining of hTERT-HPNE, BxPC3 and PANC-1 total proteins separated by 12% SDS-PAGE. Left to right; Input, elutes of the three cell lines using antibody anti-RhoGDI3; elutes of the three cell lines using an unrelated antibody. (B) The immunoprecipitates were then subjected to Western blotting of immunoprecipitated RhoGDI3 protein on protein G showing a specific band in the input and in the immunoprecipitation, nor in unrelated antibody. (C) The membrane was stripped and confronted with antibody anti-RhoG. The cells were lysed in buffer containing 50 mM Tris (pH 6.8), NaCl 2M and Triton X-100 1%.
(TIFF)

S2 Fig RhoB recruits RhoGDI3 in hTERT-HPNE pancreatic cell line. Lysates of hTERT-HPNE cell line was immunoprecipitated (IP) with anti-RhoB and unrelated antibody, (A) Coomassie blue staining of hTERT-HPNE total protein separated by 12% SDS-PAGE. Left to right; MW, Input, elutes of the cell line using antibody unrelated and anti-RhoB antibodies; unbinding unrelated protein and unbinding anti-RhoB protein; wash unrelated and anti-RhoB beads. (B) The immunoprecipitates were then subjected to Western blotting of immunoprecipitated RhoB protein on protein G showing a specific band in the input and in the immunoprecipitation, nor in unrelated antibody. (C) The membrane was stripped and confronted with antibody anti-RhoGDI3. The cells were lysed in buffer containing 50 mM Tris (pH 6.8), NaCl 2M and Triton X-100 1%.
(TIFF)

S3 Fig Phase contrast micrographs of BxPC3, to show the patch growth of this cell line. BxPC3 is a cell line derived from PDAC with no evidence of metastasis. It is evident the growth of this cell line in clusters.
(TIFF)

S4 Fig The normal pancreatic tissue samples showed a strong RhoGDI3 immunoreactivity in the different type of cells: pancreatic islets (arrowheads) and ducts (arrows) (A), whilst, RhoG, showed an immunoreactivity pattern very low or absent, pancreatic islets (arrowheads) and ducts (arrows) (B). Scale bar 100 μm.
(TIFF)

S5 Fig RhoGDI3 is not localized neither in the nuclei of BxPC3 nor in the nuclei of PANC-1 cell lines. After cells were treated with rhEGF (depicted above the images as 0, 2 and 10 rhEGF Min) nuclear (N) and cytosolic (c) fractions from BxPC3 (A) and PANC-1 (B), cells were obtained and analyzed by immunoblotting, using anti-RhoGDI3, anti-RhoG, anti-RhoB antibodies. Anti-histone H3 antibody was used as a nuclear control and anti-Aldolase B antibody as a cytosol control. 20 μg of cell lysates were loaded. Membranes were overexposed for 1 min to evidence all the bands.
(TIFF)

S6 Fig The localization of RhoGDI3 in hTERT-HPNE and PANC-1 pancreatic cell lines. Cells were starved 6 hours and confronted with rhEGF for the period of 0, 2 and 10 minutes (Marked as 0, 2 and 10 rhEGF min). A) To show the cytoskeleton reorganization, F-Actin was stained with rhodamine phalloidin (red), and (B) fluorescence microscopic staining of RhoGDI3 (green) were carried out in hTERT-HPNE (left column), and PANC-1 (right column). The time point of 2 min and 10 min show the detail of RhoGDI3 staining to highlight the signal at the lamellipodial protrusions evident only in the cell lines hTERT-HPNE and PANC-1 (white arrowheads), not in BxPC3 cells (Data not shown). Scale bar 100 μm for panel A and 10 μm for panel B.
(TIF)
