# Supplementary figures and images for: Immunological and Functional Characterization of RhoGDI3 and Its Molecular Targets RhoG and RhoB in Human Pancreatic Cancerous and Normal Cells
Source: PLoS One. 2016 Nov 10;11(11):e0166370. doi: 10.1371/journal.pone.0166370 (PMC5104321; doi:10.1371/journal.pone.0166370)

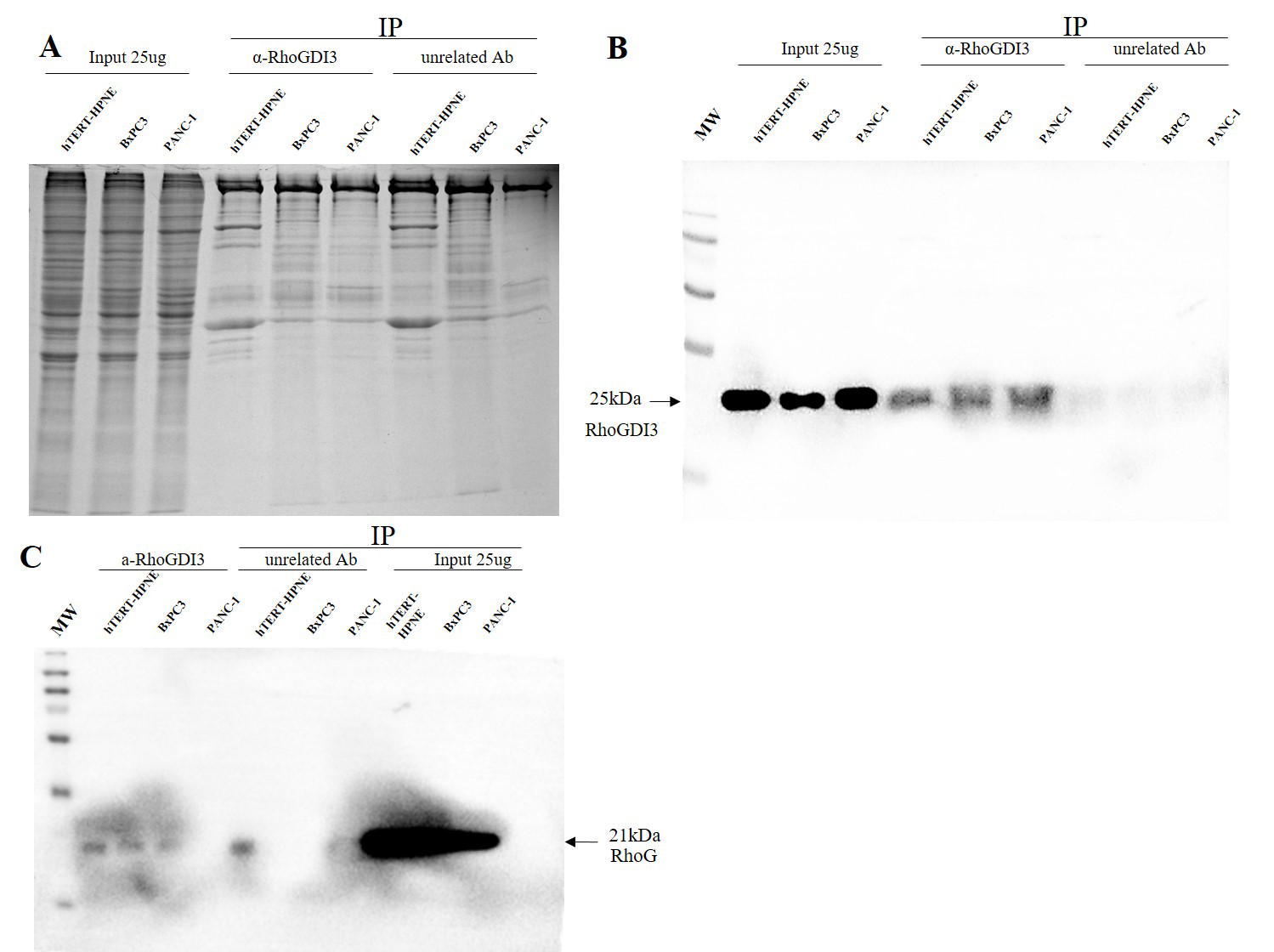

Supplement: S1 Fig — Lysates from the three cell lines were immunoprecipitated (IP) with anti-RhoGDI3 and unrelated antibody, (A) Coomassie blue staining of hTERT-HPNE, BxPC3 and PANC-1 total proteins separated by 12% SDS-PAGE. Left to right; Input, elutes of the three cell lines using antibody anti-RhoGDI3; elutes of the three cell lines using an unrelated antibody. (B) The immunoprecipitates were then subjected to Western blotting of immunoprecipitated RhoGDI3 protein on protein G showing a specific band in the input and in the immunoprecipitation, nor in unrelated antibody. (C) The membrane was stripped and confronted with antibody anti-RhoG. The cells were lysed in buffer containing 50 mM Tris (pH 6.8), NaCl 2M and Triton X-100 1%. (TIFF) [file pone.0166370.s001.tiff]

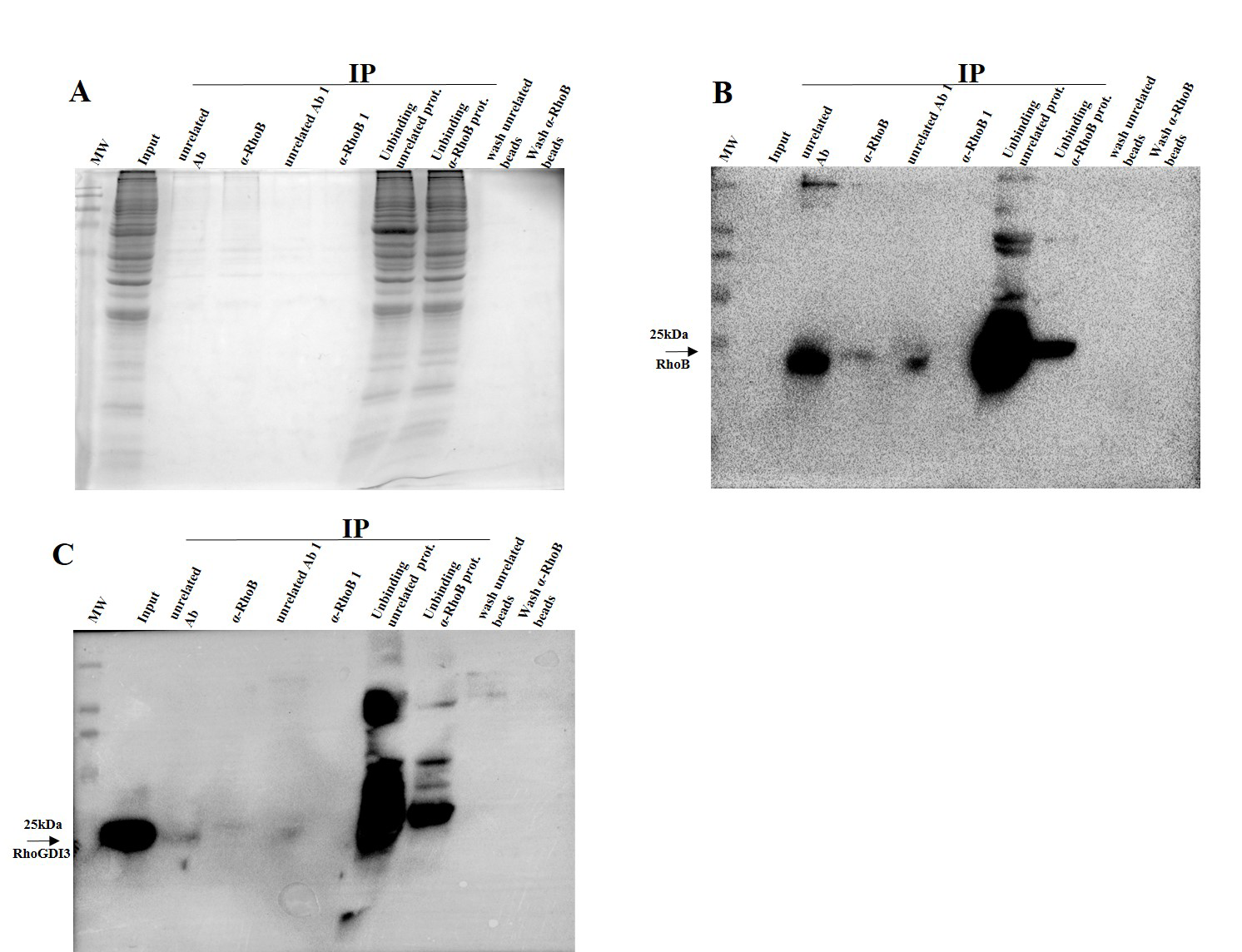

Supplement: S2 Fig — Lysates of hTERT-HPNE cell line was immunoprecipitated (IP) with anti-RhoB and unrelated antibody, (A) Coomassie blue staining of hTERT-HPNE total protein separated by 12% SDS-PAGE. Left to right; MW, Input, elutes of the cell line using antibody unrelated and anti-RhoB antibodies; unbinding unrelated protein and unbinding anti-RhoB protein; wash unrelated and anti-RhoB beads. (B) The immunoprecipitates were then subjected to Western blotting of immunoprecipitated RhoB protein on protein G showing a specific band in the input and in the immunoprecipitation, nor in unrelated antibody. (C) The membrane was stripped and confronted with antibody anti-RhoGDI3. The cells were lysed in buffer containing 50 mM Tris (pH 6.8), NaCl 2M and Triton X-100 1%. (TIFF) [file pone.0166370.s002.tiff]

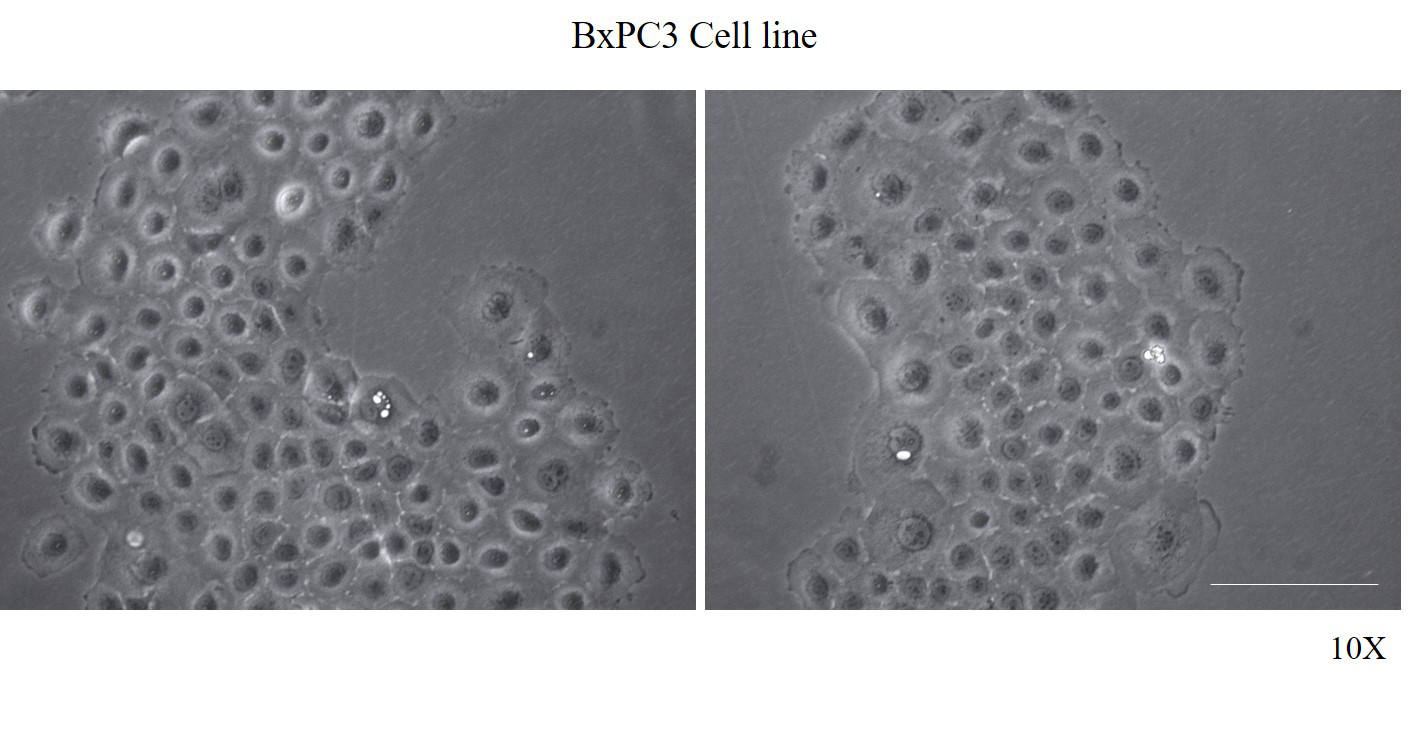

Supplement: S3 Fig — BxPC3 is a cell line derived from PDAC with no evidence of metastasis. It is evident the growth of this cell line in clusters. (TIFF) [file pone.0166370.s003.tiff]

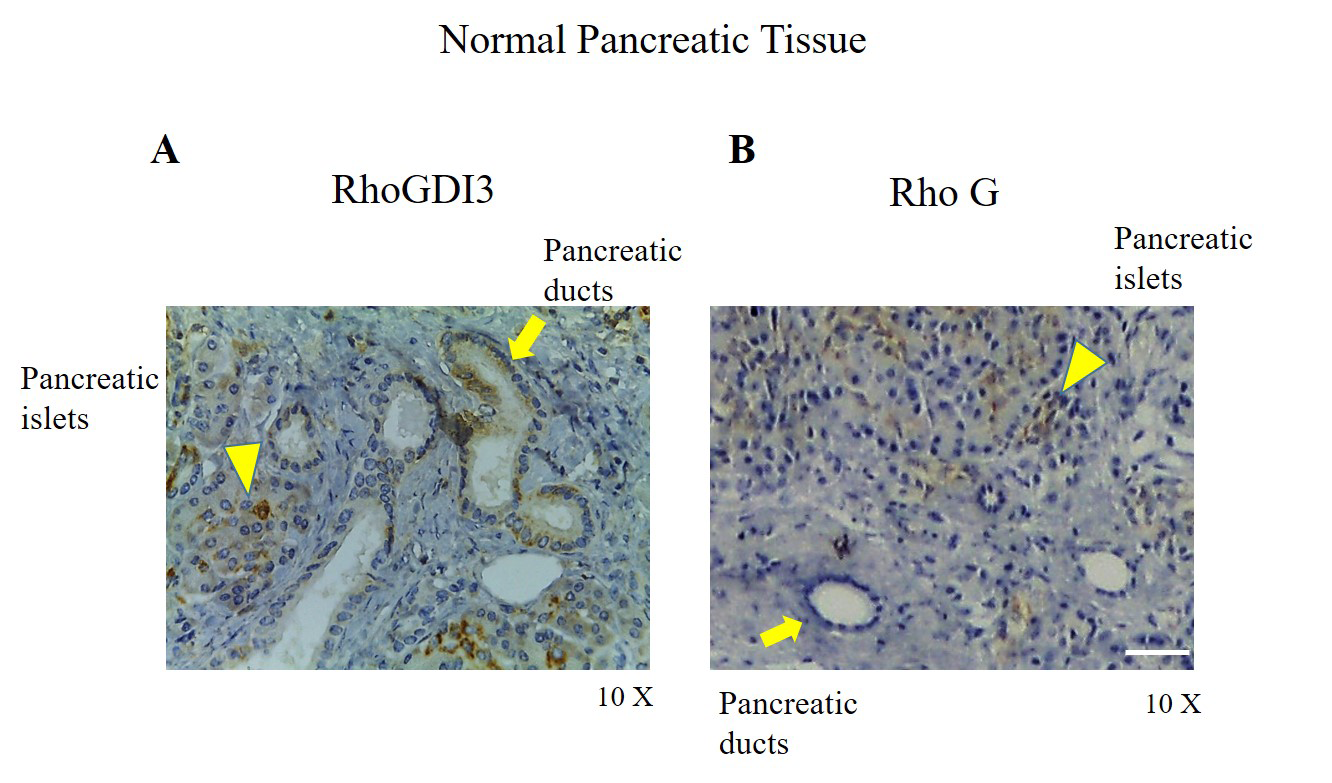

Supplement: S4 Fig — Scale bar 100 μm. (TIFF) [file pone.0166370.s004.tiff]

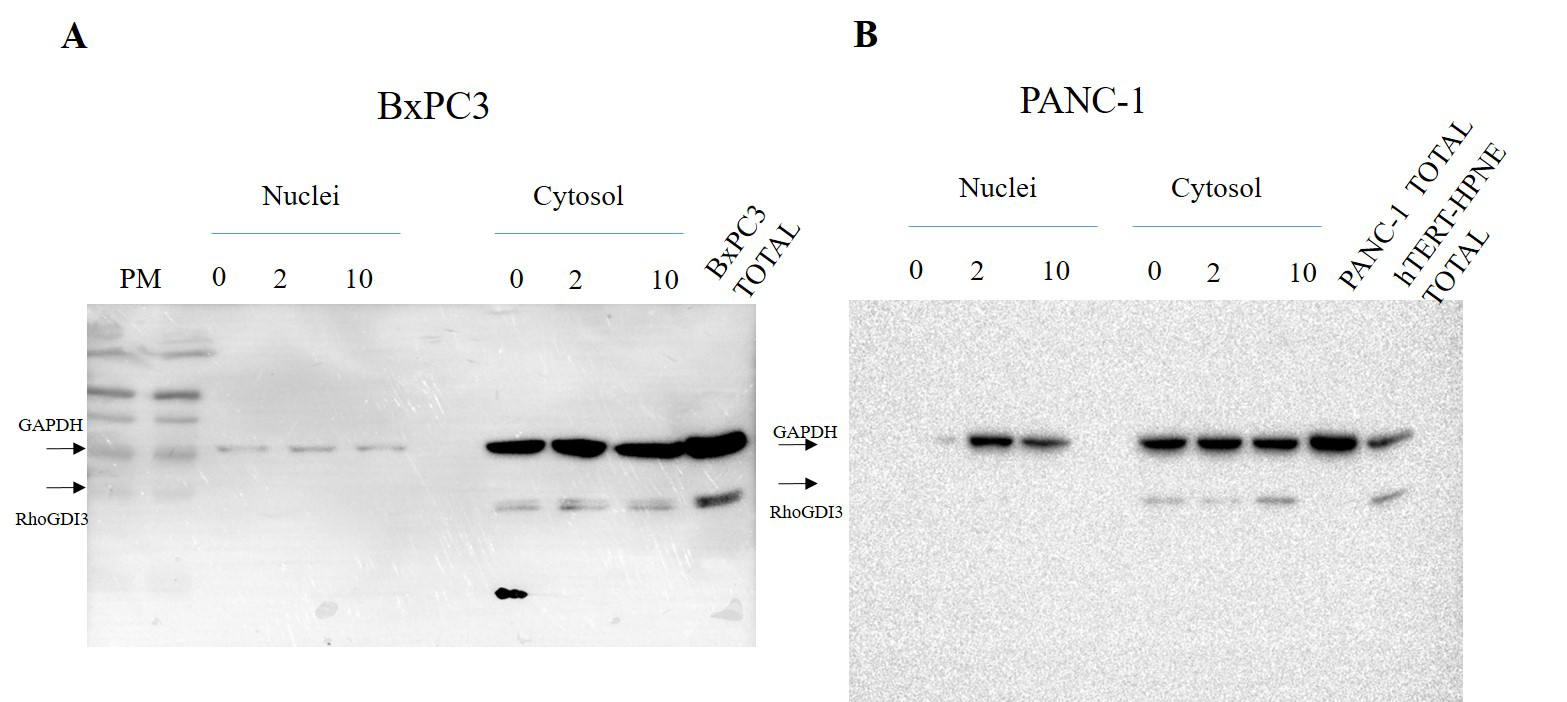

Supplement: S5 Fig — After cells were treated with rhEGF (depicted above the images as 0, 2 and 10 rhEGF Min) nuclear (N) and cytosolic (c) fractions from BxPC3 (A) and PANC-1 (B), cells were obtained and analyzed by immunoblotting, using anti-RhoGDI3, anti-RhoG, anti-RhoB antibodies. Anti-histone H3 antibody was used as a nuclear control and anti-Aldolase B antibody as a cytosol control. 20 μg of cell lysates were loaded. Membranes were overexposed for 1 min to evidence all the bands. (TIFF) [file pone.0166370.s005.tiff]

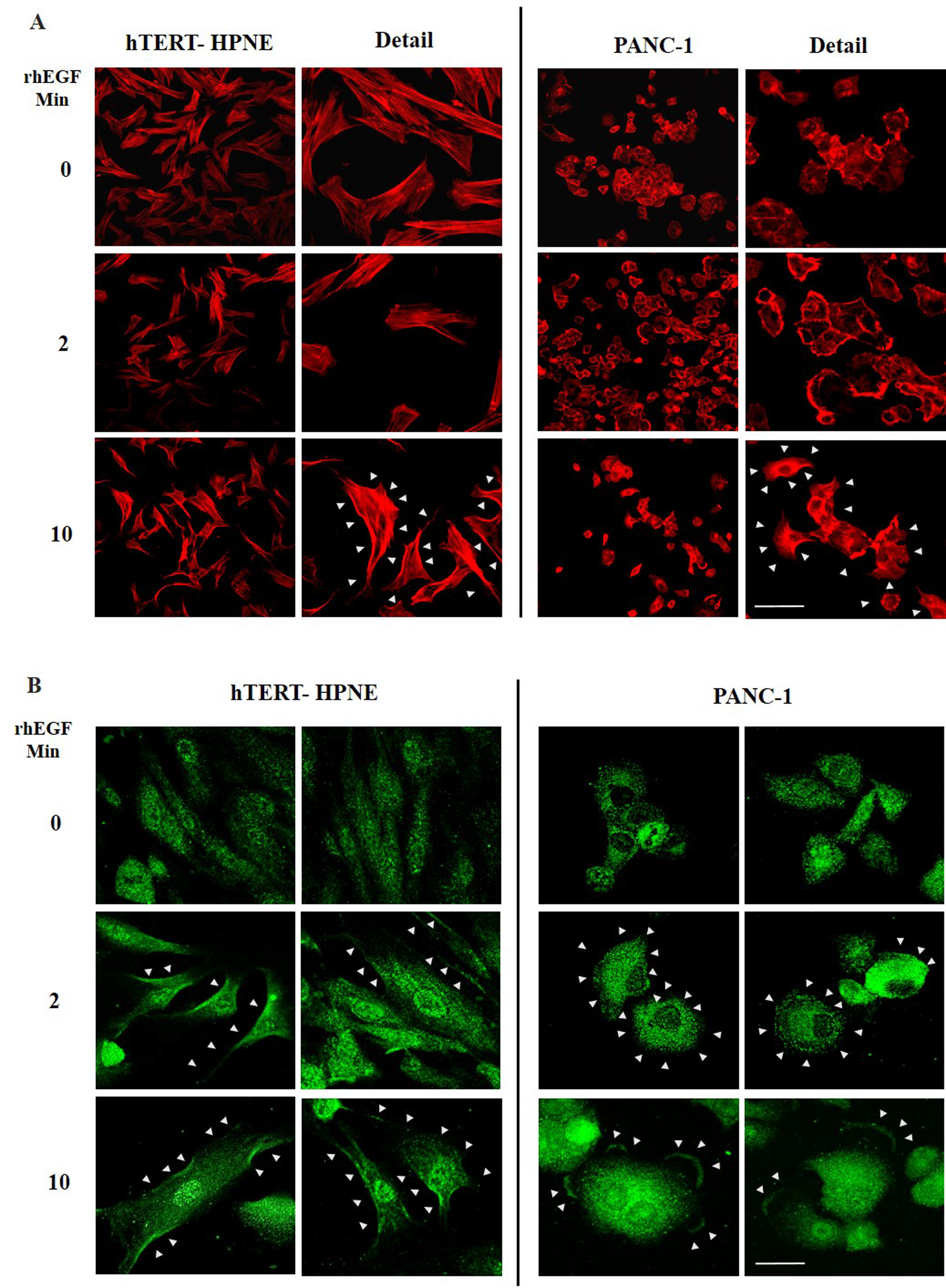

Supplement: S6 Fig — Cells were starved 6 hours and confronted with rhEGF for the period of 0, 2 and 10 minutes (Marked as 0, 2 and 10 rhEGF min). A) To show the cytoskeleton reorganization, F-Actin was stained with rhodamine phalloidin (red), and (B) fluorescence microscopic staining of RhoGDI3 (green) were carried out in hTERT-HPNE (left column), and PANC-1 (right column). The time point of 2 min and 10 min show the detail of RhoGDI3 staining to highlight the signal at the lamellipodial protrusions evident only in the cell lines hTERT-HPNE and PANC-1 (white arrowheads), not in BxPC3 cells (Data not shown). Scale bar 100 μm for panel A and 10 μm for panel B. (TIF) [file pone.0166370.s006.tif]
